# Neutrophil-to-Lymphocyte Ratio, Systemic Immune-Inflammation Index, and HALP Score as Predictors of Mortality in Acute Respiratory Distress Syndrome

**DOI:** 10.3390/jcm15114344

**Published:** 2026-06-04

**Authors:** Anwar A. Sayed, Layan A. Alrehaili, Alhanouf O. Alsuhaymi, Ethar H. Alnuzha, Ghaida T. Alsaedi, Raghad M. Alsharif, Shaden H. Alsaedi, Shatha S. Althubyani, Taif A. Alahmadi, Wurayf F. Alharbi

**Affiliations:** 1 Department of Basic Medical Sciences, College of Medicine, Taibah University, Madinah 42353, Saudi Arabia; 2 Health and Life Research Center, Taibah University, Madinah 42353, Saudi Arabia; 3 College of Medicine, Taibah University, Madinah 42353, Saudi Arabia

**Keywords:** acute respiratory distress syndrome, hematological biomarkers, neutrophil-to-lymphocyte ratio, systemic immune-inflammation index, HALP score, mortality prediction, risk stratification

## Abstract

**Background**: Acute respiratory distress syndrome (ARDS) remains one of the most serious causes of respiratory failure and mortality in critically ill patients. Although the Berlin Definition provides a standardized framework for diagnosis, it offers limited predictive value for clinical outcomes. In this context, there is growing interest in the use of routinely available hematological markers as practical tools for early risk stratification. This study aimed to examine the association between hematological parameters and mortality in patients with ARDS, with particular emphasis on complete blood count-derived inflammatory indices. **Methods**: This multicenter retrospective cohort study included 404 adult patients with a confirmed diagnosis of ARDS who were admitted to intensive care units in Saudi Arabia. Demographic, clinical, and laboratory data were collected from electronic medical records. In addition to standard hematological parameters, the neutrophil-to-lymphocyte ratio (NLR), systemic immune-inflammation index (SII), and hemoglobin–albumin–lymphocyte–platelet (HALP) score were calculated from admission laboratory findings. Comparisons between survivors and non-survivors were performed using non-parametric statistical tests, and a receiver operating characteristic (ROC) curve analysis was used to evaluate the prognostic performance of these indices for in-hospital mortality. **Results**: Of the 404 included patients, 295 survived, and 109 died during hospitalization. Non-survivors demonstrated significantly higher white blood cell and neutrophil counts, alongside significantly lower lymphocyte, eosinophil, hemoglobin, mean corpuscular hemoglobin, mean corpuscular hemoglobin concentration, and albumin levels. The derived inflammatory indices further demonstrated clear differences between outcome groups, as NLR and SII were significantly higher in non-survivors, whereas HALP scores were significantly lower. In ROC analysis, NLR showed the strongest discriminatory ability for mortality (AUC = 0.80, 95% CI 0.74–0.85), followed by SII (AUC = 0.76, 95% CI 0.71–0.81) and HALP (AUC = 0.76, 95% CI 0.70–0.81). The optimal cutoff values were 4.26 for NLR, 958 for SII, and 2.15 for HALP. No significant correlations were identified between age and any of the three indices. Upon applying multivariable regression analysis, only NLR maintained its prognostic ability for ARDS-related mortality. **Conclusions**: Routine hematological parameters, together with derived inflammatory and nutritional indices, were significantly associated with mortality in patients with ARDS. Among the evaluated markers, NLR demonstrated the strongest prognostic performance, both in univariate and multivariate analysis, followed by SII and HALP. As these indices are derived from inexpensive, readily available laboratory tests, they may offer practical value for early risk stratification and clinical decision-making, particularly in resource-limited settings.

## 1. Introduction

Acute Respiratory Distress Syndrome (ARDS) remains one of the most formidable challenges in critical care medicine, representing a leading cause of respiratory failure and mortality among critically ill patients worldwide [1]. ARDS is characterized by diffuse alveolar damage, increased pulmonary vascular permeability, and severe hypoxemia. ARDS affects approximately 10% of intensive care unit (ICU) admissions and carries a mortality rate ranging from 35% to 46% despite advances in supportive care [2]. The syndrome’s heterogeneous nature, coupled with its rapid progression and variable clinical presentation, necessitates early identification of high-risk patients and timely implementation of appropriate therapeutic interventions to improve outcomes. The cornerstone of ARDS diagnosis is the Berlin criteria [3], which classify the syndrome based on timing, chest imaging findings, origin of pulmonary edema, and severity of hypoxemia. While these criteria have standardized ARDS recognition globally [4], they primarily serve diagnostic purposes and offer limited prognostic value for individual patient risk stratification. Current clinical assessment methods predominantly depend on conventional radiological and physiological parameters, which, although useful for initial diagnosis, often fail to capture the full spectrum of disease severity [5]. This limitation is particularly critical in healthcare settings, where early identification of patients at the highest risk of adverse outcomes could significantly influence treatment intensity and clinical decision-making.

The pathophysiology of ARDS involves a complex interplay of inflammatory mediators, immune cell activation, and systemic responses that extend beyond the pulmonary compartment [6]. Emerging evidence suggests that systemic inflammation, as reflected in hematological parameters, may serve as a window into disease severity and progression [7]. Similar to other respiratory infections, routine blood tests, such as the complete blood count (CBC)—including white blood cell counts, platelet levels, hemoglobin concentrations, and other readily available hematological markers—are performed universally in ICU settings and may offer valuable prognostic information [8]. However, despite their accessibility and cost-effectiveness [9], the systematic evaluation of these parameters as biomarkers for ARDS severity stratification remains limited and fragmented in the literature.

Among the readily available hematological parameters derived from routine Complete Blood Count (CBC) tests, several composite biomarkers have emerged as promising indicators of systemic inflammation and immune response in critically ill patients. The Neutrophil-to-Lymphocyte Ratio (NLR) reflects the balance between innate and adaptive immune responses, with elevated ratios indicating heightened inflammatory states and immune dysregulation [10]. The Systemic Immune-Inflammation Index (SII), calculated from platelet, neutrophil, and lymphocyte counts, provides a comprehensive assessment of the inflammatory and immune status of critical illness [11]. Additionally, the Hemoglobin, Albumin, Lymphocyte, Platelet (HALP) score combines markers of nutritional status, immune function, and hemostasis to reflect the disease burden in patients [12]. These composite indices, derived from standard laboratory tests, offer the potential to capture complex pathophysiological processes through simple, reproducible calculations that can be implemented immediately at the bedside without requiring additional testing or specialized equipment.

The existing literature comprises predominantly single-center studies with small sample sizes, heterogeneous patient populations, and variable methodological approaches, limiting the generalizability and clinical applicability of findings. To address this critical gap, this multicenter, retrospective study investigates the association between routinely available complete blood count-derived biomarkers, specifically the neutrophil-to-lymphocyte ratio (NLR), systemic immune-inflammation index (SII), and hemoglobin, albumin, lymphocyte, and platelet (HALP) score, and key clinical outcomes, i.e., mortality. By analyzing comprehensive data across multiple hospitals, this study seeks to enhance the generalizability of its findings while focusing on readily accessible markers in routine clinical practice. The identification of reliable hematological markers may improve risk stratification and inform clinical decision-making in ARDS, particularly in healthcare settings with limited access to advanced diagnostic modalities.

## 2. Materials and Methods

### 2.1. Study Design and Setting

This was a retrospective cohort study, designed and conducted across multiple centers. It included adult patients diagnosed with acute respiratory distress syndrome (ARDS) who were admitted to intensive care units in several hospitals in Saudi Arabia. The study included 404 patients admitted to the hospital with a confirmed diagnosis of acute respiratory distress syndrome, as defined by the Berlin Definition (2012), to standardize diagnosis and classify the condition’s severity [3].

Briefly, ARDS is an acute-onset respiratory failure that develops within a week of a known clinical insult or worsening respiratory symptoms. Radiologically, it is characterized by bilateral pulmonary opacities on chest imaging that cannot be fully explained by nodules, lung collapse, or pleural effusion, and respiratory failure that cannot be fully explained by fluid overload or cardiac failure. The PaO_2_/FiO_2_ ratio is used to further categorize ARDS severity, with a minimum positive end-expiratory pressure (PEEP) of 5 cm H_2_O. ARDS severity could be divided into three categories: mild (PaO_2_/FiO_2_ 200–300 mmHg), moderate (100–200 mmHg), and severe (<100 mmHg) [13].

### 2.2. Participants and Eligibility Criteria

The study population included all adult patients (≥18 years) who were admitted to the intensive care unit during the study period with a confirmed diagnosis of acute respiratory distress syndrome. Patients were identified from ICU admission records, electronic ARDS diagnostic codes, and clinical notes to ensure all criteria were met.

The criteria for patients’ inclusion were as follows:

1. If they were aged 18 years or older.
2. If they had a confirmed diagnosis of acute respiratory distress syndrome (ARDS) according to the Berlin Definition (2012). Eligible patients were required to have severe respiratory failure meeting the Saudi Ministry of Health intensive care unit admission criteria and to have a complete blood count (CBC) available at admission or at the time of ARDS diagnosis.
3. These criteria indicate that symptomatic patients may need to be admitted if they have acute respiratory distress syndrome, oxygen saturation below 94% on room air, or clinical or radiological signs of pneumonia.

Patients’ exclusion criteria were as follows:

1. Those with serious comorbid conditions that raise the risk of serious medical conditions, such as chronic kidney disease, diabetes mellitus, hypertension, and cardiovascular disease.
2. Morbid obesity with a body mass index of ≥40.
3. Active cancer.
4. A history of organ transplantation or other immunosuppressive conditions, like the use of biological immunosuppressive drugs, may also need to be admitted. Clinical judgment may also be used to admit individuals with additional co-occurring conditions that call for inpatient care [14].
5. Patients were excluded from the study if essential laboratory or demographic data were missing.
6. If an ARDS diagnosis could not be reliably confirmed according to previously stated criteria, or if the medical records were duplicates, incomplete, or insufficient for analysis, patients were excluded.

### 2.3. Data Collection and Laboratory Procedures

The study used a retrospective review of medical records from the hospital’s electronic system. Researchers used a standardized data collection form to ensure uniformity across centers. Detailed patient data variables were collected (Table S1). NLR: The neutrophil-to-lymphocyte ratio (NLR) was computed using the complete blood count (CBC) data. For each patient, the NLR was calculated as the absolute neutrophil count divided by the absolute lymphocyte count [13].

The systemic immune-inflammatory index (SII) was computed as the ratio of the product of platelet and neutrophil counts to the lymphocyte count [15].

The formula used to calculate the HALP score was hemoglobin (g/L) multiplied by albumin (g/L) and lymphocyte count (/L), then divided by the platelet count (/L) [16].

### 2.4. Ethical Considerations

The study was conducted in accordance with the Declaration of Helsinki and the health research standards in the Kingdom of Saudi Arabia. The study received approval from the Ethics Committee at Taibah University—College of Medicine.

Because the study was retrospective and relied on pre-existing medical records, patients were exempted from informed consent. Confidentiality was protected by removing all identifying information and storing data in encrypted systems accessible only to the research team.

### 2.5. Statistical Analysis

Statistical analysis was performed using GraphPad Prism software (Version 10.1). As this was a retrospective cohort study, all variables were analyzed at a single time point. The distribution of continuous variables was assessed using a Shapiro–Wilk normality test, which showed that the data were not normally distributed. Accordingly, continuous variables were presented as medians with interquartile ranges (IQRs), while categorical variables were summarized as frequencies and percentages.

Due to the non-parametric distribution of the data, comparisons between ‘patients’ subsets were conducted using the Mann–Whitney U test for continuous variables. Comparisons between more than 2 groups were conducted using the Kruskal–Wallis Test, with Dunn’s correction for multiple comparisons.

To assess the diagnostic performance of the Neutrophil-to-Lymphocyte Ratio (NLR), Systemic Immune-Inflammation Index (SII), and Hematological Assessment of Lymphocyte–Platelet (HALP) score for predicting in-hospital mortality, Receiver Operating Characteristic (ROC) curves were constructed. To address potential overfitting and provide optimism-corrected estimates of the Area Under the Curve (AUC), internal validation was performed using bootstrap resampling with 500 iterations. Optimism-corrected median AUCs, along with their corresponding 95% confidence intervals (CIs), were calculated. All internal validation procedures were executed using the “pROC” package [17] in R software (version 4.3.0) [18]. The optimal cutoff value for each parameter was determined using the Youden index [19,20], which identifies the threshold that maximizes the sum of sensitivity and specificity. Cutoff values were calculated separately for NLR, SII, and HALP based on the study cohort. The comparison between the AUCs was conducted using DeLong’s test [21].

Multivariable logistic regression analysis was performed to identify independent predictors of in-hospital mortality. Variables entered into the model included NLR, SII, HALP, and [any other adjusted covariates, such as Age]. Odds ratios (OR) and their corresponding 95% confidence intervals (CIs) were calculated. To assess for potential multicollinearity among the highly related immune-inflammatory indices, variance inflation factors (VIF) were computed for all covariates. All logistic regression analyses were conducted using the base stats package and the “car” package [22] in R. Patients with missing data were excluded from the analysis.

Associations between inflammatory indices and age were examined using Spearman’s rank correlation coefficient and non-parametric correlation analysis. A *p*-value < 0.05 was considered statistically significant.

## 3. Results

### 3.1. Participant Characteristics

A total of 404 patients were included in this retrospective cohort study. The median age of the study population was 38 years (interquartile range 28–57.75). Among the patients, 197 were male (48.76%), and 207 were female (51.23%). According to hospital records, 295 patients (73.01%) were discharged alive, whereas 109 patients (26.98%) died during hospitalization. These characteristics are described in Table 1.

### 3.2. Laboratory Overall Characteristics of the Study Participants

The hematological and biochemical characteristics of the research participants are compiled in Table 2.

The median RBC count, as measured by red blood cell indices, was 4.71 × 10^6^/mL (IQR: 4.36–5.07). The median hematocrit was 40.35% (IQR: 37.20–43.90), and the median hemoglobin level was 13.30 g/dL (IQR: 11.99–14.60). In addition, 86.90 fL (IQR: 82.23–90.28), 28.90 pg (IQR: 26.90–30.20), and 33.500 g/L (IQR: 32.10–34.00) were the median mean corpuscular volume (MCV), mean corpuscular hemoglobin (MCH), and mean corpuscular hemoglobin concentration (MCHC), respectively. Meanwhile, 223.50 × 10^3^/mL was the median platelet count (IQR: 179.30–277.00). Regarding white blood cell parameters, the median WBC count was 5.76 × 10^3^/mL (IQR: 4.27–7.98). The median counts of neutrophils and lymphocytes were 1.17 × 10^3^/mL (IQR: 0.77–1.69) and 3.59 × 10^3^/mL (IQR: 2.42–5.78), respectively. The median eosinophil and monocyte counts were 0.04 ×10^3^/mL (IQR: 0.01–0.09) and 0.31 × 10^3^/mL (IQR: 0.23–0.45), respectively. The median albumin level among the biochemical parameters was 43.00 g/L (IQR: 36.00–50.75).

### 3.3. Comparison of Laboratory Parameters Between Survivors and Non-Survivors

Regarding erythrocyte parameters, non-survivors had significantly lower hemoglobin levels (*p* = 0.0076), and mean corpuscular hemoglobin (MCH) (*p* = 0.0219). No statistically significant differences were observed in red blood cell count, hematocrit, or mean corpuscular volume (MCV) and mean corpuscular hemoglobin concentration (MCHC) between survivors and non-survivors. Additionally, platelet counts were comparable between the two groups (*p* = 0.9552). Non-survivors exhibited significantly higher white blood cell counts (*p* < 0.0001) and neutrophil levels (*p* < 0.0001) compared with survivors. In contrast, lymphocyte counts were significantly lower among non-survivors (*p* < 0.0001). Similarly, eosinophil counts were markedly reduced in non-survivors (*p* < 0.0001), whereas monocyte counts did not differ significantly between the two groups (*p* = 0.9984). Serum albumin levels were significantly lower in non-survivors compared with survivors (*p* < 0.0001). These results are summarized in Table 3.

### 3.4. Comparisons of NLR, SII, and HALP Between Survivors and Non-Survivors

**Figure 1:**
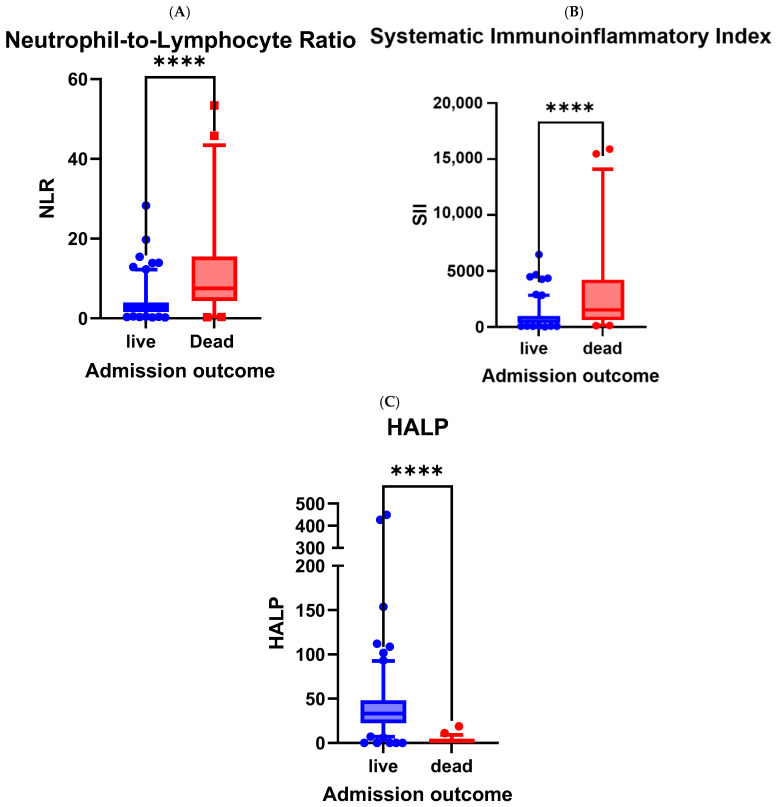
Box plots illustrate the comparison of (**A**) neutrophil-to-lymphocyte ratio (NLR), (**B**) systemic immune-inflammation index (SII), and (**C**) hemoglobin–albumin–lymphocyte–platelet (HALP) according to admission outcomes (survivors vs. non-survivors). Both NLR and SII were significantly higher in non-survivors compared to survivors, whereas HALP levels were significantly lower in non-survivors. These findings indicate a strong association between these inflammatory markers and patient outcomes, with statistically significant differences observed between the two groups. **** *p* value < 0.0001.

As the median values of most hematological parameters in both survivors and non-survivors were within the reference ranges, composite inflammatory markers were analyzed to better differentiate clinical outcomes. One of these markers is the neutrophil-to-lymphocyte ratio (NLR), which depends on both neutrophil and lymphocyte counts and reflects the balance between inflammatory and immune responses (1). Comparison of NLR values between survivors and non-survivors demonstrated that non-survivors had significantly higher NLR levels at admission than survivors (*p* < 0.0001; Figure 1A).

Another established hematological biomarker is the Systemic Immune-Inflammation Index (SII), which is calculated as the product of the platelet count and the neutrophil-to-lymphocyte ratio (NLR). When comparing the two groups, SII values were significantly higher among non-survivors than survivors (*p* < 0.0001; Figure 1B), further confirming the strong association between increased inflammatory activity and mortality risk.

In addition to inflammatory markers, a composite nutritional–inflammatory indicator, the hemoglobin–albumin–lymphocyte–platelet (HALP) score, was also evaluated. The HALP score incorporates hemoglobin and albumin concentrations, as well as lymphocyte and platelet counts, reflecting both nutritional status and immune function. Comparison of HALP scores between the two groups revealed significantly lower HALP values than among survivors at admission (Figure 1C), suggesting that poorer nutritional and immune status may also be associated with unfavorable clinical outcomes.

### 3.5. The Receiver Operating Characteristic (ROC) Curve in Discriminating Between Survivors and Non-Survivors

**Figure 2:**
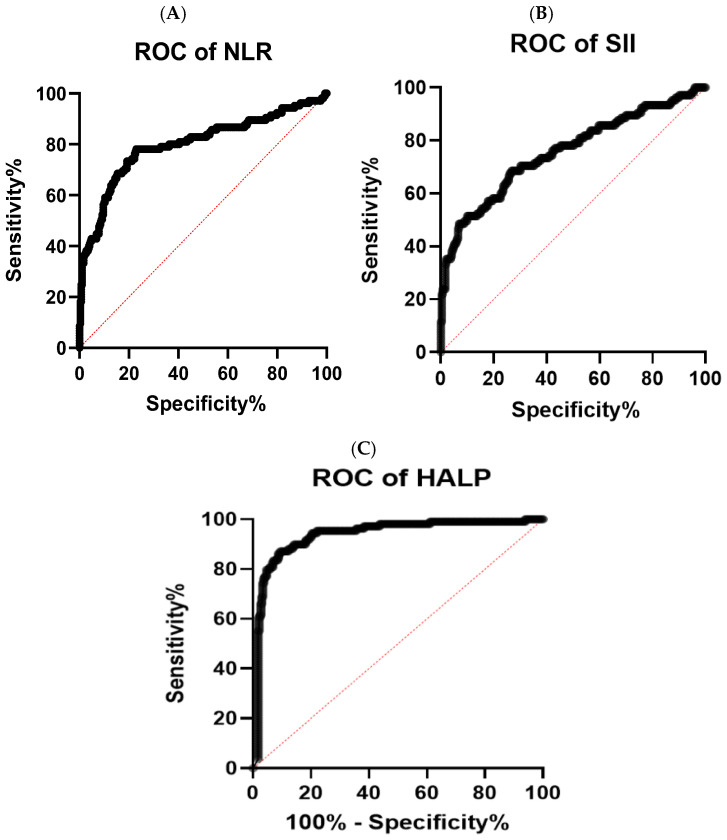
Receiver operating characteristic (ROC) curves demonstrated the predictive ability of (**A**) neutrophil-to-lymphocyte ratio (NLR), (**B**) hemoglobin–albumin–lymphocyte platelet (HALP), and (**C**) systemic immune-inflammation index (SII) for mortality. All three parameters demonstrate good discriminatory ability, with their curves lying above the reference line, indicating their effectiveness in distinguishing between survivors and non-survivors.

Receiver operating characteristic (ROC) curve analysis was performed to evaluate the ability of the neutrophil-to-lymphocyte ratio (NLR), systemic immune-inflammation index (SII), and hemoglobin–albumin–lymphocyte platelet (HALP) parameters to differentiate between survivors and non-survivors and to predict mortality risk (Figure 2). The ROC curves for all three parameters remained consistently above the reference line, indicating that each one of them can reliably distinguish between the survivors and non-survivors.

For NLR (Figure 2A), the AUC was 0.80 (95% CI: 0.74–0.85; *p* < 0.0001), indicating significant predictive accuracy. The best cutoff value for NLR was 4.26, which yielded a sensitivity of 78.10% (95% CI: 69.27–84.94%), specificity of 77.40% (95% CI: 72.26–81.82%), and a likelihood ratio of 3.455.

The SII also demonstrated good discriminative capacity (Figure 2B), with an AUC of 0.76 (95% CI: 0.69–0.82; *p* < 0.0001). The best cutoff value for SII was 958, yielding a sensitivity of 68.57% (95% CI: 58.18–75.81%), specificity of 73.29% (95% CI: 67.93–78.04%), and a likelihood ratio of 2.53.

For HALP (Figure 2C), the AUC was 0.76 (95% CI: 0.70–0.81; *p* < 0.0001), indicating excellent discriminatory ability. The best cutoff value for HALP was 2.15, which yielded a sensitivity of 64.76% and a specificity of 79.31%, with a likelihood ratio of 4.29 (Tables S2 and S3).

To evaluate whether the differences in the diagnostic accuracy of the three hematological indices were statistically meaningful, pairwise comparisons of their ROC curves were conducted using DeLong’s test. The results demonstrated that the prognostic performance of the NLR was significantly superior to that of the SII (DeLong’s Z = 2.56, *p* < 0.01). However, the discriminative capacity of NLR did not differ significantly from that of the HALP score (Z = 1.40, *p* = 0.16). Similarly, no statistically significant difference in diagnostic accuracy was observed between SII and HALP (Z = 0.06, *p* = 0.95; Table S4).

### 3.6. NLR, SII, HALP Are Independent Parameters of Age

**Figure 3:**
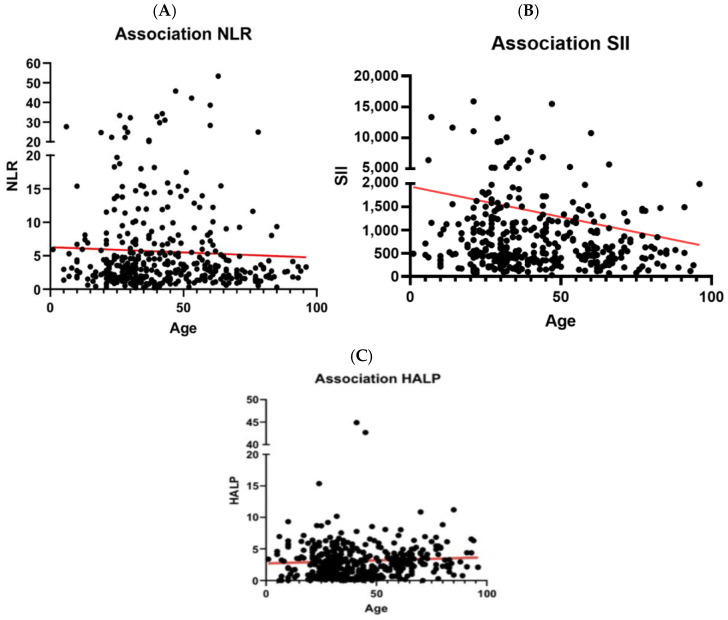
Correlation analysis to test for association between (**A**) NLR and Age. Correlation analysis to test for association between (**B**) SII and Age, (**C**) HALP and Age. Red lines represent regression lines which demonstrate the correlation trend between the different variables.

Correlation analysis showed no significant correlation between NLR and age (r = −0.037, *p* = 0.460). The scatter plot shows a random distribution with an almost flat trend line. The wide range of results across age groups demonstrates that NLR is age-independent (Figure 3A).

Correlation analysis revealed no statistically significant association between SII and age (r = 0.08, *p* = 0.871). Although a slight negative relationship existed, the association was not statistically significant, as the SII values were widely scattered across all ages (Figure 3B).

There was no clear relationship between age and HALP score (r = 0.071, *p* = 0.153). The scatter plot showed a random distribution with an almost flat trend line, indicating that HALP is age-independent (Figure 3C).

**Figure 4:**
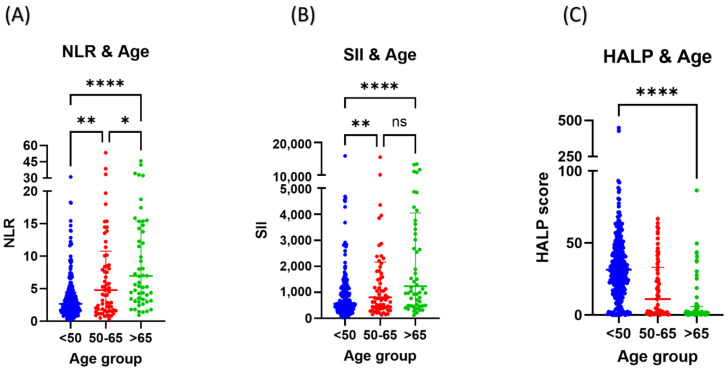
Dot plots demonstrating comparisons of NLR, SII, and HALP scores based on patients’ age. The dot plots show comparisons between those aged younger than 50 years (blue), between 50 and 65 years (red), and older than 65 years (green). The plots show comparisons of (**A**) NLR, (**B**) SII, and (**C**) HALP scores. HALP: Hemoglobin, Albumin, Lymphocyte, and Platelet score; NLR: Neutrophil-to-Lymphocyte Ratio; ns: Not statistically Significant; SII: Systemic Immune-Inflammatory Index. * *p* value < 0.05; ** *p* value < 0.01; **** *p* value < 0.0001.

Interestingly, the relationship between age and these markers, NLR, SII, and HALP, was not linear. As demonstrated in Figure 3, age did not correlate significantly with any of these markers. However, upon dividing the patients’ cohort into 3 subgroups based on their age, younger than 50, 50–65 years old, and older than 65 years, statistically significant changes were observed (*p* values < 0.0001). NLR was highest among those aged 65 years or older, both compared with those younger than 50 (*p* value < 0.0001; Figure 4A) and with those aged 50–65 (*p* value < 0.05; Figure 4A). Similarly, the SII was highest among those aged 65 years or older compared with those aged 50 years or younger (*p* value < 0.0001; Figure 4B). On the other hand, those younger than 50 years had the highest HALP score compared with the other subgroups (*p* value < 0.0001; Figure 4C).

To determine the prognostic value of these indices for mortality, a multivariate regression analysis was performed (Table S5). In multivariate analysis, NLR (OR = 1.22, [1.12–1.34], *p* < 0.0001) was associated with higher rates of mortality. HALP (OR = 0.90, [0.77–1.03], *p* = 0.16), SII (OR = 1.0, [0.99; 1.0], *p* = 0.78) were not associated with the rate of mortality outcome (Table 4). To evaluate potential multicollinearity among the overlapping hematological indices (NLR, SII, and HALP) included in the multivariable logistic regression model, collinearity diagnostics were performed. The Variance Inflation Factors (VIF) for all adjusted covariates were well below the conservative threshold of 5.0 (NLR: 2.23, SII: 2.48, and HALP: 1.24). These findings demonstrate the absence of significant multicollinearity, confirming that the calculated odds ratios and associated confidence intervals are statistically stable and robust.

In our univariate ROC analyses, NLR, SII, and HALP all demonstrated statistically significant areas under the curve (AUC), indicating that each baseline hematological index holds crude prognostic value for predicting in-hospital mortality in ARDS patients. However, when these indices were evaluated simultaneously within a multivariable logistic regression model, only NLR retained independent statistical significance.

## 4. Discussion

In this retrospective study, differences in routinely measured hematological parameters between survivors and non-survivors with ARDS were examined. Mortality, an unfortunate yet expected outcome of ARDS, was also observed in our study cohort. Almost 27% of our cohort did not survive the condition. While such a percentage could be considered low, previous studies have indicated that ARDS-associated mortality ranges between 11 to 87% [23]. Interestingly, mortality decreased over the years at a rate of 1.1%/year [24]. While our cohort excluded patients with comorbidities, such as diabetes and cancer, conditions that would contribute to and lead to death, patients who had sepsis, on top of ARDS, showed significantly lower mortality figures, ranging between 11.6 and 18.1% [25]. Such discrepancies further underscore the complexity of ARDS pathophysiology and the need for additional mechanistic studies.

We found that white blood cell and neutrophil counts in non-survivors were significantly higher than those in survivors, consistent with the fact that severe ARDS is characterized by an extremely intense systemic inflammatory response [26,27]. Neutrophils play a major role in the pathogenesis of ARDS, as they are excessively recruited and activated in the lungs, then release proteolytic enzymes and reactive oxygen species that disrupt the alveolar–capillary barrier and worsen pulmonary edema [7,26]. Previous studies have consistently reported that increased neutrophil counts are associated with disease severity and mortality, making them useful prognostic markers that are easily obtainable in critically ill ARDS patients [27].

On the other hand, non-survivors had significantly lower lymphocyte counts, suggesting a failure of the adaptive immune response in the advanced stage of disease. Lymphopenia has been shown to correlate with greater disease severity and poorer clinical outcomes in ARDS; thus, immune dysregulation is considered the underlying factor rather than a primary hematologic abnormality [28]. A retrospective study showed that decreased lymphocyte counts are associated with higher mortality and longer ICU stays, especially when associated with neutrophilia. This emphasizes the prognostic significance of combined immune imbalance in ARDS [29].

Similarly, non-survivors had significantly lower eosinophil counts. Peripheral eosinopenia has been increasingly reported as a severe systemic inflammatory and stress response marker in ARDS [30]. Large cohort studies have shown that high eosinophil counts are independently associated with lower mortality; on the other hand, persistently low eosinophil levels are more commonly seen in non-survivors, suggesting a more severe inflammatory phenotype [31]. The above data demonstrate that eosinophil depletion is a factor in the severity of the condition rather than a direct eosinophil-related pathology.

Monocyte counts did not show any statistically significant difference between survivors and non-survivors. This means that the absolute monocyte count alone may not be sufficient to distinguish different outcomes in ARDS. On the other hand, a prior study found that composite indices, such as the monocyte-to-lymphocyte ratio, better reflect immune imbalance and are strongly correlated with increased short-term mortality [32]. This indicates that monocyte-related prognostic information is clinically relevant primarily in the context of lymphocyte suppression.

Regarding erythrocyte indices, in our study, non-survivors demonstrated significantly lower hemoglobin, mean corpuscular hemoglobin, and mean corpuscular hemoglobin concentration, whereas red blood cell count, hematocrit, and mean corpuscular volume did not differ significantly between survivors and non-survivors.

Anemia is a feature frequently observed in ARDS and critically ill patients, mainly resulting from inflammation, erythropoietic suppression, and altered iron metabolism rather than acute hemorrhage [33]. The direct evidence of hemoglobin level association with ARDS mortality is still quite limited; however, anemia can worsen tissue hypoxia and thus, indirectly contribute to the progression of organ failure in severe diseases [34].

In our cohort, platelet counts were comparable between survivors and non-survivors. This is in direct contradiction with what has been previously reported in ARDS. ARDS, when associated with thrombocytopenia, is thought to demonstrate increased platelet activation and consumption within the pulmonary microvasculature, in addition to systemic inflammatory effects [35]. Previous studies have consistently reported an association between thrombocytopenia, elevated mortality, and greater disease severity in ARDS [36]. Besides their role in hemostasis, platelets are involved in inflammatory and immune processes, and high platelet–neutrophil interactions may further worsen endothelial damage and cause deterioration of gas exchange [26]. Such a contradiction may be attributed to the study design, which obtained a single platelet count observation at a time when the disease is in its early stages. Later stages, which may lead to patient mortality, might be preceded by thrombocytopenia.

Serum albumin levels were noticeably lower in the non-survivors. Hypoalbuminemia is commonly observed in those patients who are critically ill and mainly represents systemic inflammation and increased vascular permeability rather than only an indication of nutritional deficiency [37]. Data from critically ill adult populations have shown a direct correlation between decreased serum albumin levels and the increased likelihood of developing ARDS, thus corroborating serum albumin as a marker of disease severity and inflammatory load rather than a direct cause of death [38].

This study evaluated the prognostic value of the neutrophil-to-lymphocyte ratio (NLR) in distinguishing survivors from non-survivors. The NLR comparison was chosen because the mean values of most hematological indicators in both survivors and non-survivors were within the reference ranges. Non-survivors had a significantly higher NLR at hospital admission than survivors, consistent with Song et al., who demonstrated a significant difference in the lymphocyte-to-neutrophil ratio (LRN) between survivors and non-survivors of acute respiratory distress syndrome (ARDS) [39]. Similarly, Hong and colleagues showed similar results in which non-survivors had significantly higher NLR compared to survivors, and this was associated with mortality [40]. Similar results were also previously reported in other respiratory infections, such as COVID-19 [13]. Although Song et al. assessed the lymphocyte-to-neutrophil ratio LRN rather than the neutrophil-to-lymphocyte ratio NRL, their findings are biologically consistent with ours. This supports the role of this ratio as an indicator of poor prognosis; both studies confirm that an imbalance between neutrophils, reflecting the innate immune system, and lymphocytes, representing the adaptive immune system, is strongly associated with adverse outcomes. Such an immunological imbalance system demonstrates the lack of targeted immune response against pathogens and generalized inflammation.

The results of this study demonstrated the predictive value of the neutrophil-to-lymphocyte ratio (NLR) in survival analysis, and it is largely age-independent when assessed linearly. However, our findings were consistent with those of Ming’s study [41], which demonstrated that the NLR ratio increases significantly with age, with older adults (over 65 years) exhibiting higher NLR values than younger adults (18–65 years). These results are consistent with the good predictive performance observed in both the ROC curve and the multivariate logistic regression analyses, indicating that NLR is a strong predictive biomarker.

In this study, we also evaluated the predictive value of the systemic inflammatory response index (SII) for distinguishing survivors from non-survivors. SII levels were significantly higher in patients who did not survive, confirming a strong association between elevated inflammation levels and mortality risk. These results are consistent with Pan et al.’s findings [41], who demonstrated that elevated levels of SII are associated with increased mortality in patients with acute respiratory distress syndrome (ARDS). In our cohort, the optimal SII threshold was set to exceed 959 based on receiver operating characteristic (ROC) curve analysis, whereas Pan’s study used a higher threshold (SII ≥ 1694). Nevertheless, SII was found to be an age-independent predictive biomarker. These findings are consistent with our previous discussion that the interaction between the innate (represented by neutrophils) and adaptive (represented by lymphocytes) arms of the immune system with platelets is dynamic and reflects disease activity.

Regarding HALP, our study showed that survivors had a higher HALP score than non-survivors. This was confirmed by another study by Zhang et al., which reported that more than 20,000 non-survivors had lower HALP scores than survivors [42]. In their study, HALP was a significant predictor of mortality, with a cutoff of HALP score < 3.2. Furthermore, our study’s findings that the HALP score was associated with higher mortality were consistent with previous studies. Uluc and colleagues demonstrated that patients with lower HALP scores had lower mortality rates [43]. However, the cutoff HALF score suggested for predicting mortality was 9.97. Such a discrepancy could be explained by differences in sample size and patient characteristics. The sample size in Zhang’s study was over 20,000 patients, yielding a score comparable to those in our studies. On the other hand, Uluc’s study included 405 patients, very close to our sample size; however, all were 65 years of age or older.

This study evaluated the predictive performance of three hematological indices—NLR, SII, and HALP—for predicting disease severity and mortality. Our results showed that all three indices had good discriminatory power for predicting mortality, with AUCs of 0.79, 0.75, and 0.93 for NLR, SII, and HALP, respectively. This indicates that HALP and NLR showed slightly better predictive performance than SII. Elevated NLR and SII values in non-survivors reflect a severe inflammatory response characterized by neutrophilia and lymphopenia, while low hemoglobin–albumin–lymphocyte–platelet ratios in non-survivors reflect the combined effect of systemic inflammation. These findings are consistent with previous studies that evaluated inflammatory markers for predicting COVID-19 disease severity and mortality [15]. In that study, both the NLR and SII were significantly elevated in non-survivors compared to survivors and proved useful biomarkers for predicting disease severity and mortality. Nevertheless, NLR showed better predictive performance than SII, and our findings are consistent with this.

It is important to note that the clinical value of these indicators extends beyond their predictive power alone. They are also useful due to their ease of use. For example, in our research, we used the neutrophil-to-lymphocyte ratio (NLR), the systemic inflammation index (SII), and the HALP index, all derived from routine laboratory tests and therefore inexpensive and readily available. Therefore, our study supports the use of these indicators as practical tools for early risk and disease assessment and prognosis prediction, particularly in resource-limited settings [9].

The strengths of the study include that it is the first of its kind to examine and compare multiple hematological parameters in patients with acute respiratory distress syndrome in relation to disease severity and mortality. This comprehensive analysis provides a good understanding of the inflammatory and immunological responses associated with outcomes. In addition, we used an adequate sample size, which enhances the reliability of the results and strengthens comparisons between survivors and non-survivors. These factors strengthen the study and support the clinical significance of the observed effects.

### 4.1. Limitations of the Study

However, despite these strengths, the study has several limitations that should be carefully considered when interpreting the findings. First, the cohort design precludes any inference of causality between disease severity and hematological parameters; the reported associations reflect statistical relationships only and may be confounded by unmeasured factors such as disease duration, treatment intensity, or coexisting conditions. Second, including participants across a broad age range without stratification into narrower age groups limits the ability to detect age-specific patterns, as hematological values vary substantially with age. Third, all laboratory measurements were obtained from a single time point, which cannot capture intra-individual variability due to diurnal fluctuations, transient physiological states, or dynamic changes during the disease course, thereby reducing the precision of the estimated associations. An additional constraint is the lack of detailed data on medications, comorbidities, and nutritional status, which could influence both hematological indices and disease severity and thus represent unaddressed confounders.

Finally, the external validity of our findings must be carefully considered, given our highly selected study population. The cohort’s young median age (38 years) and the strict exclusion of major global drivers of ARDS mortality, such as diabetes, hypertension, chronic kidney disease, cardiovascular disease, obesity, malignancy, and chronic immunosuppression, yielded a uniquely comorbidity-free sample. This strict filtering explains the relatively preserved baseline laboratory values observed at admission, including normal ranges for hemoglobin, albumin, and total white blood cell counts, which sharply contrast with the highly deranged baselines typically seen in older, multi-morbid ARDS populations globally. While this selection effect serves as a methodological strength by isolating the acute prognostic signals of NLR, SII, and HALP from the confounding chronic systemic inflammation of pre-existing diseases, it inevitably limits direct generalizability to routine clinical practice in regions with older or more morbid demographic profiles. In such populations, competing risks of mortality from chronic organ failure may overshadow the acute hematological shifts captured by these indices. These geographic and demographic selection effects represent a clear limitation, and our specific cut-off thresholds should be validated in broader, unselected, and heterogeneous ARDS populations before widespread clinical adoption.

### 4.2. Recommendations for Future Research

To build on the present findings, we recommend that future studies employ prospective longitudinal designs with repeated measurements of hematological parameters at clinically relevant intervals. Such an approach would allow for the assessment of temporal trajectories and help establish temporality. Age should be divided into predefined, clinically meaningful categories (e.g., pediatric, adult, and older adult groups) to enable more accurate age-related analyses. In addition, we suggest that the studied hematological indicators be combined with validated clinical scoring systems, such as the Sequential Organ Failure Assessment (SOFA) [44], the Acute physiology and chronic health evaluation (APACHE II) [45], or the Charlson Comorbidity Index [46].

## 5. Conclusions

This retrospective cohort study demonstrated that routine hematological parameters and inflammatory indices are significantly associated with mortality in patients with acute respiratory distress syndrome (ARDS). Non-survivors showed higher white blood cell and neutrophil counts and lower lymphocyte, hemoglobin, and albumin levels, reflecting severe systemic inflammation. Composite biomarkers including the neutrophil-to-lymphocyte ratio (NLR), systemic immune-inflammation index (SII), and hemoglobin–albumin–lymphocyte–platelet (HALP) score, also showed prognostic value. Higher NLR and SII and lower HALP scores were associated with increased mortality risk. However, only NLR was found to be consistently prognostic of ARDS-related mortality. As these markers are derived from routine laboratory tests, they represent accessible and cost-effective tools for early risk stratification. However, the retrospective design and single-time measurements limit causal inference. Future prospective and multicenter studies are required to validate these findings. Their integration into clinical assessment may enhance prognostic evaluation and support timely decision-making in the management of ARDS.

## Figures and Tables

**Table 1:** Patients’ Characteristics.

Characteristic	Values
Age (years)	38 (28–57.75) *
Gender	Male: 197 (48.76%)
	Female: 207 (51.23%)
Admission outcome	Survivors: 295 (73.01%)
	Non-survivors: 109 (26.98%)

* Age was described in median (interquartile range).

**Table 2:** Laboratory overall characteristics of the study participant.

Characteristic	Median (IQR)
RBC count (×10^6^/mL)	4.71 (4.36–5.07)
Hemoglobin (g/dL)	13.30 (11.99–14.60)
Hematocrit (%)	40.35 (37.20–43.90)
MCV (fl)	86.90 (82.23–90.28)
MCH (pg)	28.90 (26.90–30.20)
MCHC (g/dL)	33.50 (32.10–34.00)
Platelet count (×10^6^/mL)	223.50 (179.30–277.00)
WBC count (×10^3^/mL)	5.76 (4.27–7.98)
Neutrophil count (×10^3^/mL)	3.59 (2.42–5.78)
Lymphocyte count (×10^3^/mL)	1.17 (0.77–1.69)
Monocyte count (×10^3^/mL)	0.31 (0.230–0.45)
Eosinophil count (×10^3^/mL)	0.04 (0.01–0.09)
Albumin (g/L)	43.00 (36.00–50.75)

RBC, red blood cell; WBC, white blood cell; MCV, mean corpuscular volume; MCH, mean corpuscular hemoglobin; MCHC, mean corpuscular hemoglobin concentration; IQR, interquartile range.

**Table 3:** Comparison of patients’ characteristics and lab investigations between survivors and non-survivors.

Characteristic	Survivors	Non-Survivors	*p*-Value *
RBC count (×10^6^/mL)	4.72 (4.38–5.06)	4.60 (4.09–5.08)	0.2957
Hemoglobin (g/dL)	13.40 (12.30–14.80)	12.88 (11.60–14.35)	0.0076
Hematocrit (%)	40.70 (37.50–44.10)	39.70 (35.70–43.30)	0.0821
MCV (fl)	87.00 (82.80–90.40)	86.70 (80.90–89.90)	0.5077
MCH (pg)	29.00 (27.20–30.30)	28.10 (26.40–29.90)	0.0219
MCHC (g/dL)	33.10 (32.30–33.90)	32.90 (31.30–34.60)	0.53
Platelet count (×10^6^/mL)	222.00 (184.00–272.00)	235.000 (160.90–285.00)	0.9552
WBC count (×10^3^/mL)	5.19 (4.04–6.70)	8.330 (5.770–11.600)	<0.0001
Neutrophil count (×10^3^/mL)	3.17 (2.22–4.64)	6.16 (4.16–9.86)	<0.0001
Lymphocyte count (×10^3^/mL)	1.27 (0.88–1. 8)	0.76 (0.53–1.25)	<0.0001
Monocyte count (×10^3^/mL)	0.31 (0.23–0.43)	0.33 (0.20–0.53)	0.9984
Eosinophil count (×10^3^/mL)	0.05 (0.02–0.09)	0.02 (0.00–0.06)	<0.0001
Albumin (g/L)	46.00 (39.00–53.00)	35.00 (27.5044.000)	<0.0001

RBC, red blood cell; WBC, white blood cell; MCV, mean corpuscular volume; MCH, mean corpuscular hemoglobin; MCHC, mean corpuscular hemoglobin concentration. Data was described as medians (interquartile range). * *p* value was based comparisons using Mann–Whitney U test.

**Table 4:** Multivariate logistic regression analysis of the hematology-derived indices.

	Adjusted Odds Ratio [95% Confidence Interval]	*p*-Value	VIF
Intercept	0.147 [0.072–0.2852]	<0.0001 ****	-
NLR	1.22 [1.12–1.34]	<0.0001 ****	2.23
SII	1 [0.999; 1]	0.78	2.48
HALP	0.90 [0.77; 1.03]	0.16	1.24

HALP: Hemoglobin, Albumin, Lymphocyte and Platelet; NLR: Neutrophil-to-Lymphocyte Ratio; SII: Systemic Immunoinflammatory Index; VIF: Variance inflation factor. **** *p* < 0.0001.

## Data Availability

Data supporting the findings of this study are available upon reasonable request from the corresponding author.

## Supplementary Materials

The following supporting information can be downloaded at https://www.mdpi.com/article/10.3390/jcm15114344/s1, Table S1: Data Collection Variables., Table S2: Parameter’s Uncorrected AUC and their optimist-corrected AUC and 95% CI, Table S3: Scores optimal Cutoff and their optimist-corrected AUC, Table S4: Results of the AUC comparisons using DeLong’s test, Table S5: Results of the multivariate regression analysis.
