# Supplementary material for: Neutrophil-to-Lymphocyte Ratio, Systemic Immune-Inflammation Index, and HALP Score as Predictors of Mortality in Acute Respiratory Distress Syndrome
Source: J Clin Med. 2026 Jun 4;15(11):4344. doi: 10.3390/jcm15114344 (PMC13258175; doi:10.3390/jcm15114344)
Supplement: Supplementary file 1 [file jcm-15-04344-s001.zip › jcm-4307166-supplementary.pdf]

Table S1. Data Collection Variables.

1.Demographic Variables	Age
	Gender
2.Clinical Data	ARDS severity
	Need for mechanical or supportive ventilation
	Admission outcome
	Discharge outcome
3.Complete Blood Count (CBC) Parameters	Red blood cell (RBC) count
	Hemoglobin level
	Platelet count
	White blood cell (WBC) count
	Differential count: Absolute neutrophil and lymphocyte count
	Red cell distribution width (RDW)
4. Derived Hematological Indices	Neutrophil-to-lymphocyte ratio (NLR) (Neutrophil count / Lymphocyte count)
	Systemic immune-inflammatory index (SII) (Neutrophil count × platelet count/ Lymphocyte count)
	HALP index (Hemoglobin concentration × Albumin × Lymphocytes count ÷ Platelet count)

Table S2. Parameter's Uncorrected AUCs and their optimist-corrected AUCs and 95% CIs.

**Parameter**	**Uncorrected AUC**	**Uncorrected P value**	**Optimism-Corrected AUC**	**Optimism-Corrected 95% CI**
**NLR**	0.80	*<0.0001*	*0.80*	*0.74 - 0.85*
**SII**	0.76	*<0.0001*	*0.76*	*0.71 - 0.81*
**HALP**	0.76	<0.0001	0.76	0.70 - 0.81

Table S3. Scores' optimal Cutoffs and their optimist-corrected AUCs.

**Parameter**	**Optimal Cutoff (Youden)**	**Sensitivity (%)**	**Specificity (%)**
**NLR**	*4.26*	*78.10%*	*77.59%*
**SII**	*958*	*68.57%*	*73.44%*
**HALP**	*2.15*	*64.76%*	*79.31%*

Table S4. Results of the AUC comparisons using DeLong's test.

	**DeLong's Z-statistic**	**P value**
**NLR vs SII**	2.56	<0.01
**NLR vs HALP**	1.40	0.16
**SII vs HALP**	0.06	0.95

Table S5. Results of the multivariate regression analysis.

	**Estimate**	**Std.E**	**Z value**	**P value**
**(intercept)**	-1.9200020	0.3570209	-5.378	7.54e-08
**NLR**	0.2015621	0.0444844	4.531	5.87e-06
**SII**	0.0000375	0.0001369	0.274	0.784
**HALP**	-0.1089794	0.0775080	-1.406	0.160

Std.E: standard error; *Z value*, Wald *z* statistic. *Estimate* represents the unstandardized logistic regression coefficient (log-odds). Statistical significance is defined as *P* value < 0.05.
